# A systematic review and meta-analysis of hybrid vs. cemented stems – which method is more optimal for revision total knee arthroplasty?

**DOI:** 10.1186/s12891-024-07389-y

**Published:** 2024-04-10

**Authors:** Yogen Thever, Sir Young James Loh, Raghuraman Raghavan, Rong Chuin Toh, Ing How Moo

**Affiliations:** Department of Orthopaedic Surgery, Changi General Hospital, 2 Simei St 3, Simei, 529889 Singapore

**Keywords:** Cemented fixation, Hybrid fixation, Revision TKA, Loosening, Re-revision

## Abstract

**Introduction:**

The number of primary and revision Total Knee Arthroplasty (TKA) cases are expected to increase in future. There are various advantages and disadvantage to employing either of the two main types of stem fixation methods – cemented or hybrid technique. This review aimed to study the most optimal fixation method for revision TKAs by comparing radiological outcomes and re-revision rates.

**Methods:**

A systematic review and meta-analysis was performed using PubMed and Cochrane Library from 2010 to identify studies explicitly comparing outcomes between cemented against hybrid fixation revision TKA techniques, with a minimum follow up of at least 24 months. A total of 8 studies was included in this review. Egger’s test and visual inspection of the funnel plot did not reveal publication bias.

**Results:**

There was no statistically significant difference in radiological failure and loosening (OR 0.79, CI 0.37–1.66, I^2^ = 29%, *p* = 0.22), all causes of re-revision (OR 1.03, CI 0.73–1.44, I^2^ = 0%, *p* = 0.56) and aseptic revision (OR 0.74, CI 0.27–2.02, I^2^ = 0%, *p* = 0.41) between cemented and hybrid techniques. Functional and pain outcomes compared between the two fixation techniques were largely similar across the studies included in this meta-analysis.

**Conclusion:**

Despite a trend favouring hybrid stems in revision TKA, current evidence revealed that radiological outcomes and re-revision rates are largely similar between cemented and hybrid fixation techniques.

## Introduction

The number of primary Total Knee Arthroplasty (TKA) and revision TKA cases are expected to increase around the world by 2050 [1]. In America alone, the number of patients younger than 65 years old requiring a primary TKA is expected to exceed 50% of the total American population in future [2] and projected to even increase by more than six times from 2005 to 2030 [3]. Consequently, the number of revision TKAs are expected to increase correspondingly as well [3, 4]. The economical cost of a revision TKA is exceedingly substantial, and commonly result in decreased function and satisfaction for patients post-operatively [5]. Bone deficiency, osteopenia and deformity after explanting primary implants make revision cases challenging for surgeons and can affect the stability of revision implants [6]. In order to achieve a higher level of implant stability, intramedullary stems are routinely used in revision setting to achieve diaphyseal fixation for adequate stability and therewith improve the survival and clinical outcomes of revision TKA [7–9].

Stems can be placed using either a fully cemented construct or a press-fit, hybrid technique whereby the femoral and tibial components are usually cemented. There are existing studies published that have described excellent results for both hybrid and cemented stems for revision TKA, respectively [10–15]. However, there is paucity and controversy in the existing literature in terms of the optimal method of fixation in these stems. There are very few studies that make direct comparisons to both techniques [6, 16]. A meta-analysis done by Wang et al. in 2015 pooled non-comparative studies describing outcomes from each individual technique, and concluded that both techniques were similar in total failures, incidence of aseptic loosening and infection rate in both the mid to long term [17]. On the contrary, a recent systematic review and meta-analysis done by Sheridan et al. in 2020 which pooled studies only making a direct comparison between cemented and hybrid stems found that hybrid stems had a significantly lower all-cause failure rate than cemented stems [18]. However, rates of aseptic loosening and radiographic failure, were found to be statistically similar although results generally trended more favorably towards hybrid stems. Thus, it is evident that even attempts at higher levels of evidence are controversial in this regard.

The aim of this study was hence to provide an updated insight and combine existing studies in the literature to explore and evaluate the optimal fixation method for stems in revision TKA, and provide clinical guidance based on the existing literature. The authors aim to compare outcomes between hybrid and cemented stems in terms of (1) radiological failure and loosening, (2) total re-revision rates for any cause and (3) incidence of aseptic re-revision.

## Methodology

### Search strategy, eligibility and study results

The Preferred Reporting Items for Systematic Reviews and Meta-Analyses (PRISMA) guidelines were strictly adhered to throughout the study. The PubMed, Cochrane Library, Embase and Web of Science database were used to perform the literature search with the following Medical Subject Headings (MeSH) terms in different combinations: “cemented”, “uncemented”, “revision total knee”, “total knee arthroplasty”, “total knee replacement”, “outcomes”. The authors’ assessment for inclusion of studies in this review was done on two separate occasions to ensure accuracy, and ambiguity as to whether to include studies were resolved by further discussion among the authors of this paper. Final papers were also reviewed in entirety by the authors.

The authors included studies in the English language published from the year 2010 that were explicitly comparing outcomes between cemented and cementless stems directly in revision TKA. Studies describing surgical techniques, review articles, case reports and studies describing outcomes of a single technique (either use of cemented or cementless stems only) without comparison, were excluded from this review. Other inclusion criteria included a minimum follow up period of 2 years, and studies had to describe criteria surrounding the aims they were looking into as well as indications for subsequent re-revision.

**Fig. 1:**
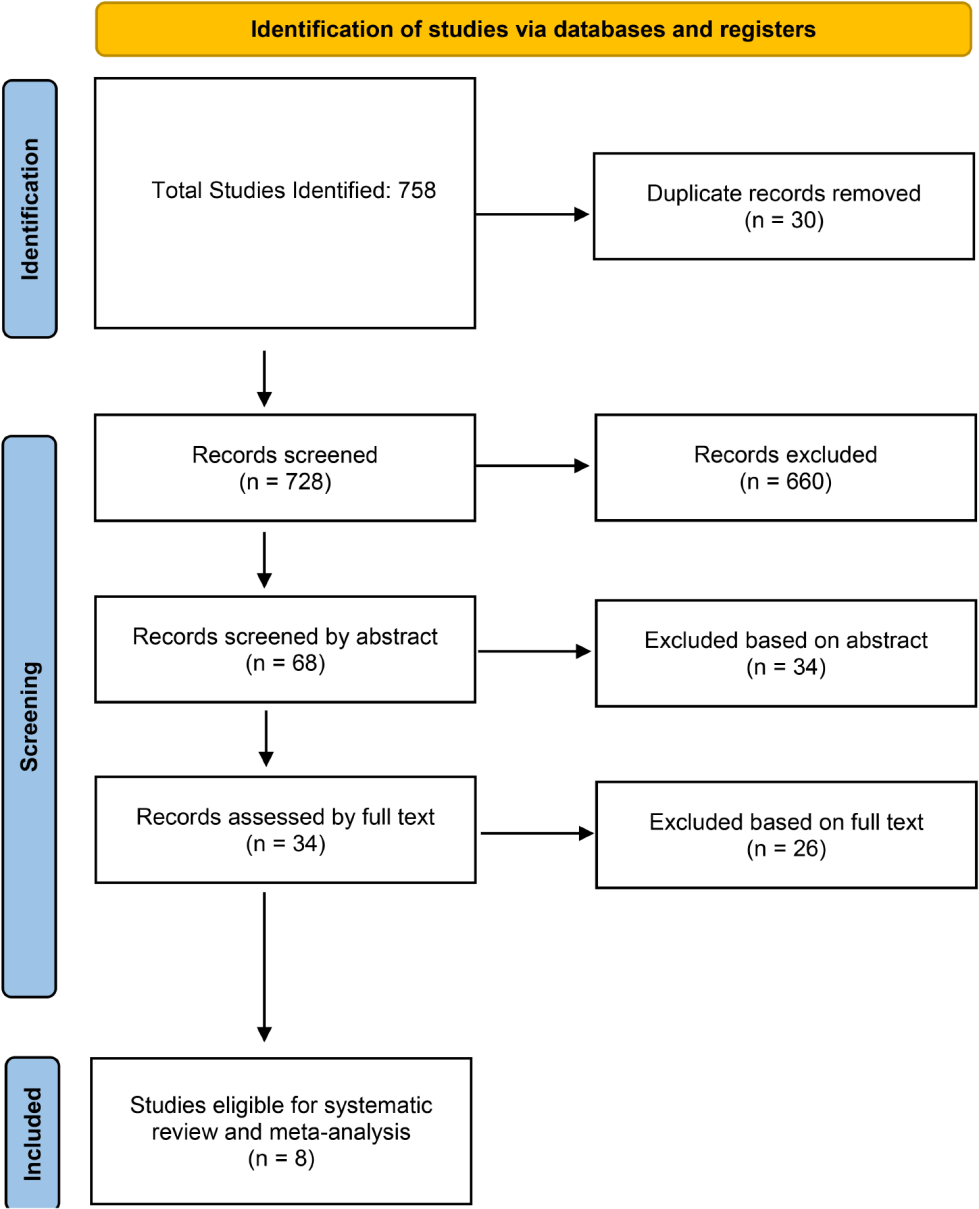
Preferred reporting items for systematic reviews and meta-analyses (prisma) flow chart

A total of 758 papers were identified for the initial screening and title screening process. 690 papers were excluded as they were not relevant to the study aims, and the remaining sixty-eight were screened again based on abstract. Thirty-four were again excluded subsequently, and the remaining thirty-four were reviewed based on full text. Eight papers were eventually selected to be included in the review, and these papers were compared against the inclusion and exclusion criteria again. The search strategy and selection are demonstrated in the PRISMA flow chart (Fig. 1).

Important information and variables relevant to this study aims were subsequently extracted and compiled. These variables included: study title, authors, journal, year, country, type of study, inclusion criteria of each respective study, total number of stems, total number of cemented and hybrid stems respectively, implants used (if reported), gender, mean age, total number of re-revisions, total number of radiological failure or loosening, total number of aseptic re-revisions cases, post-operative care regime, functional outcome, follow-up period. To address heterogeneity between studies, the authors also chose to pay particular attention to the definition of measure of each outcome to ensure it could be pooled and compared.

### Statistical analysis and bias

The meta-analysis was performed using R meta package (R version 4.2.2), which is an open-source, comprehensive statistical software, while demographic data was pooled and analyzed using descriptive statistics. The authors used a random-effects model for the meta-analysis where the odds ratio (OR) for each outcome was calculated based on a percentage weight assigned to each study. It was calculated with 95% confidence interval, where a p-value of less than 0.05 was taken to be statistically significant. Inter-study heterogeneity was assessed with the I^2^ statistic and expressed as a statistic, where a p-value of less than 0.05 was taken to be statistically significant for heterogeneity impacting the results. Publication bias was ruled out through visual inspection of a funnel plot and carrying out egger’s test.

## Results

### Summary of studies

**Fig. 2:**
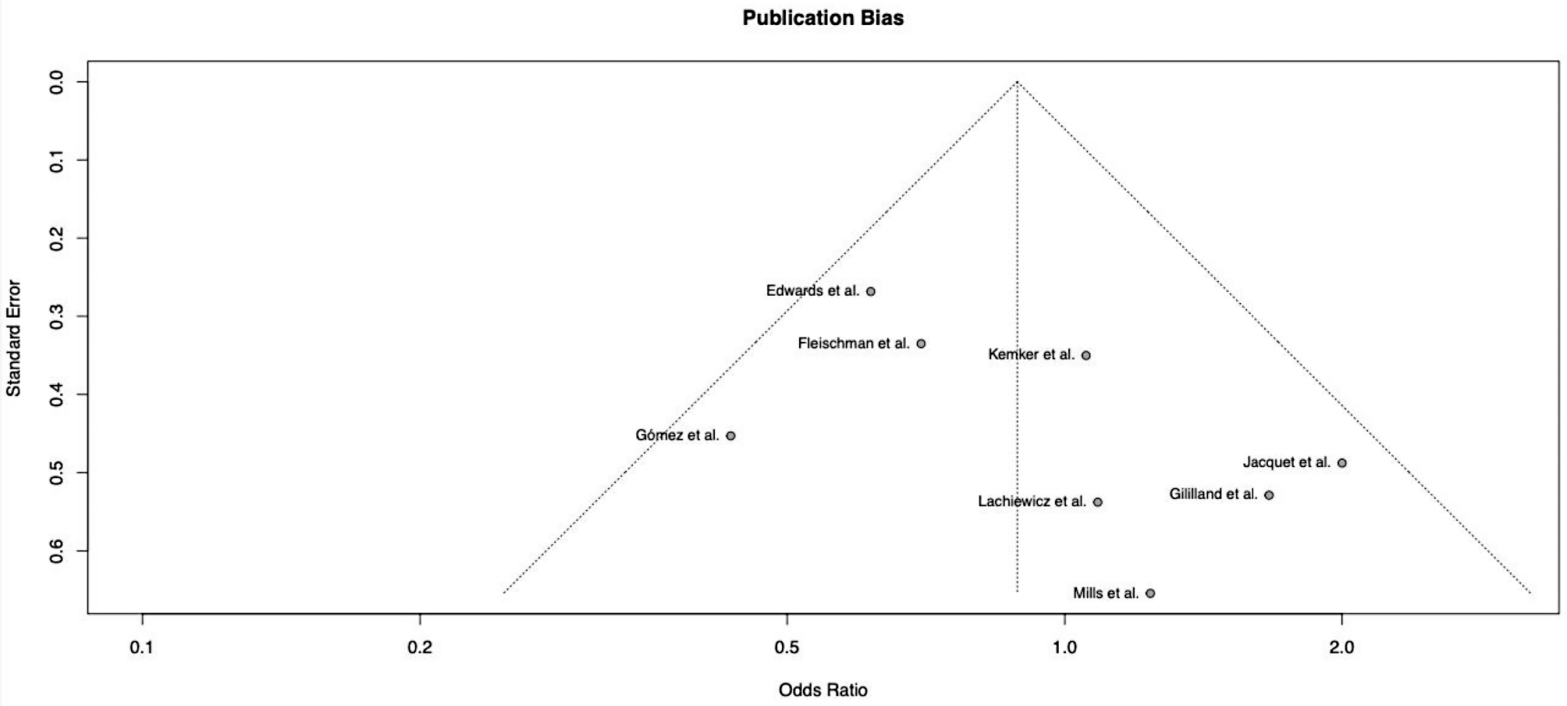
Funnel plot for papers included

Eight studies met the inclusion criteria and were included in this study (Table 1). There were a total of 1555 revision TKA cases collectively used for the comparison, of which 577 were cemented stems and 978 consisted of hybrid stems. With the available data that the authors had, the mean age of patients that received a cemented stem was 67.9 years, and that in hybrid stem was 66.9 years. Females were the majority in both cemented and hybrid groups, taking up 65.7% of patients in the cemented group, and 64.7% in the hybrid group. All studies followed up on their patients for at least 24 months and reported indications for revising their primary TKAs. In terms of indications for revision TKAs, Edwards et al. solely included only infected TKAs [16]. On the contrary, studies by Gomez et al., Gililland et al. and Jacquet et al. studied all aseptic causes of primary TKA failure [19–21]. The rest of the studies included all indications of revision TKAs including loosening, infection, instability, osteolysis or polywear, stiffness, malposition, arthrofibrosis and patellar subluxation [16, 22–25]. Majority of the studies included in this systematic review were from the United States of America (USA). There were also studies from Spain, Netherlands and France. Of note, there were no studies from Asian countries. Majority of the studies reported the implants that they opted for apart from four of the studies that did not specify [16, 20, 23, 25]. Most studies explicitly detailed their post-operative care for their patients. Lachiewicz et al. inserted drains for all the revision TKA cases done in that study, which was subsequently removed from the first post operative day, where patients were allowed to ambulate under the supervision of a physiotherapist twice a day [22]. Gomez et al. and Jacquet et al. administered low molecular weight heparin for all the cases starting from the evening before surgery, up to one month post operatively [19, 21]. Patients in those two studies as well as the study carried out by Mills et al. started ambulating immediately post-operatively [19, 21, 24]. The authors of this study visually inspected and reviewed the funnel plot for publication bias (Fig. 2) which did not reveal any significant asymmetry. Subsequently, an Egger’s regression test was performed to confirm that the studies were indeed unbiased in nature. Hence, it is evident that publication bias minimally impacted the validity of the results in this study.

**Table 1:** Summary OF studies included

Study	Year	Country	Implant Type	Mean Age		Gender		Number of Cases				Mean FU (months)	Indication for revision
				C	H	C	H	Total	C	H	Lost to FU		
Lachiewicz PF et al.	2020	USA	LCCK, Zimmer, Warsaw, IN	68.0	68.0	M 13 F 21	M 19 F 31	105	45	60	21	72	- Loosening - Infection - Instability - Osteolysis/ polywear - Pain/ stiffness - Pain/ malposition
Gómez-Vallejo J et al.	2018	Spain	Cemented: Natural Knee II, Centerpulse Warsaw, IN, USA Hybrid: P.F.C. TC-3 Sigma, DePuy Raynham, MA, USA	79.2	78.4	Collected, but not reported		134	134	58	76	0	- Aseptic loosening
Andrew N Fleischman et al.	2017	USA	Not Specified	65.8	63.9	M 33 F 75	M 135 F 181	424	108	316	0	62	- Aseptic loosening - Infection - Instability - Arthrofibrosis - Component wear - Periprosthetic fracture
Jeremy M Gililland et al.	2014	USA	Not Specified	65.0	64.0	M 24 F 25	M 11 F 21	160	98	62	0	96	- Aseptic loosening
Edwards PK et al.	2014	USA	Not Specified	65.0	65.0	M 26 F 25	M 32 F 31	228	102	126	49	45	- Septic TKA
Jacquet C et al.	2021	France	NexGen RHK Knee, Zimmer-Biomet)	72.9	72.6	Collected, but not reported		117	198	66	132	0	- Implant loosening - Pain - Polywear/osteolysis - Instability/ dislocation
Mills K et al.	2022	Netherlands	Condylar Legion revision TKA (Smith & Nephew, USA)	74.9	72.2	M 5 F 5	M 1 F 9	40	20	20	2	120	- Instability - Aseptic loosening - Component malposition/ malrotation - Patellar subluxation after malrotation - Infection - Polyethylene wear
Kemker BP et al.	2022	USA	Not Specified	63.8	63.8	M 13 F 27	M 34 F 59	266	80	186	0	25.8	- Aseptic loosening - Infection - Malalignment - Instability - Femoral fracture - Persistent pain

M = Male, F = Females, C = Cemented, H = Hybrid

### Radiological failure and loosening

Radiological failure and loosening were defined differently amongst the studies. Out of the ten studies, four [16, 19, 20, 23] defined radiological loosening using the modified Knee Society Radiographic Scoring System described by Fehring et al. [6]. This involved dividing the tibia into 6–7 zones on the anterior-posterior and lateral radiographs respectively, while the femur was evaluated in three zones on the anterior-posterior and 6–8 zones on the lateral films. Radiolucent lines were graded either partial or complete depending on the extent of it relative to the area in each zone. Apart from the four studies, Lachiewicz et al. defined radiological loosening using the conventional knee society scoring system [22]. Mills et al. defined loosening based on radiostereometric analysis scans, looking for implant micromotion of more than 1 mm translation or 1 degree rotation [24]. The remaining studies did not define radiological loosening explicitly [21, 25].

**Fig. 3:**
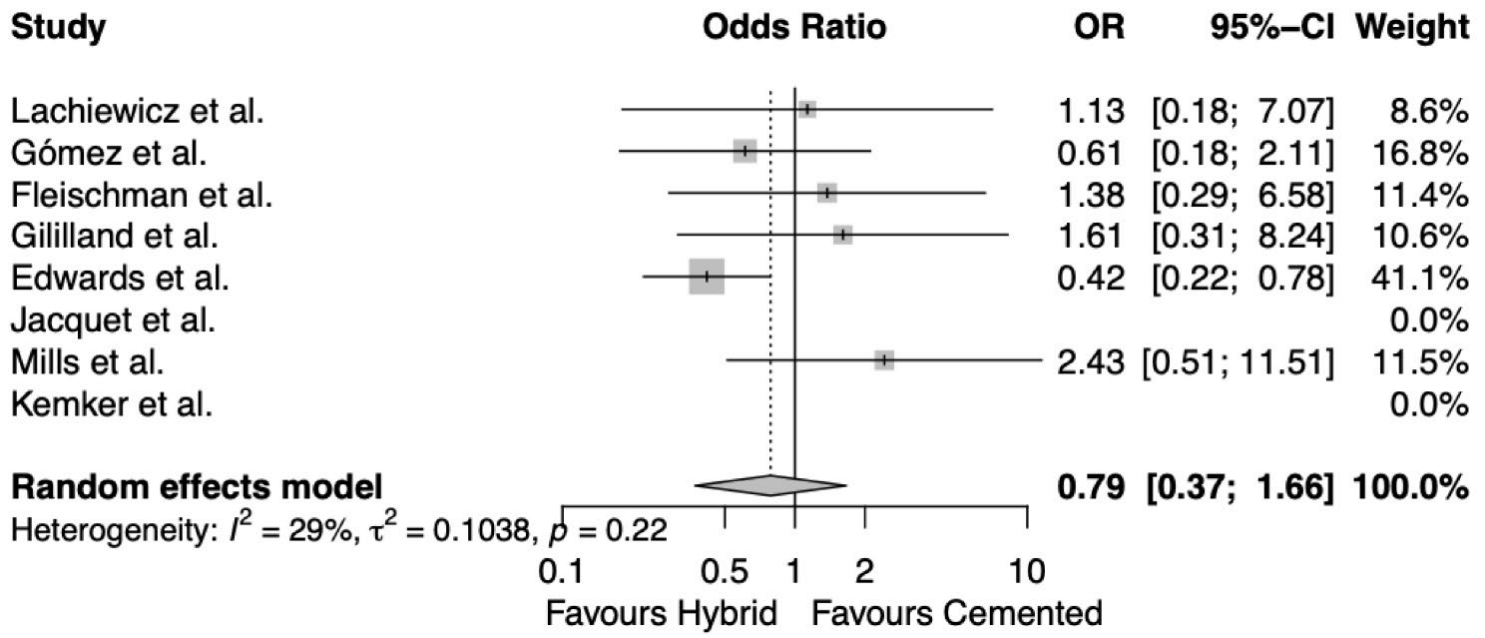
Random effects meta-analysis for radiological failure and loosening

Out of the 6 studies that investigated radiological failure and loosening, none found a statistically significant difference between cemented and hybrid stems [16, 19, 20, 22–24]. Cumulatively, there were 49 cemented stems and 46 hybrid stems that were deemed radiologically loose from the total study sample. The random effects meta-analysis (Fig. 3) revealed that there was no difference between the respective fixation methods, even though it trended favourably towards the hybrid method (OR 0.79, CI 0.37–1.66). There was no significant heterogeneity between the studies cumulatively too (I^2^ = 29%, *p* = 0.22).

### All cause re-revision

**Fig. 4:**
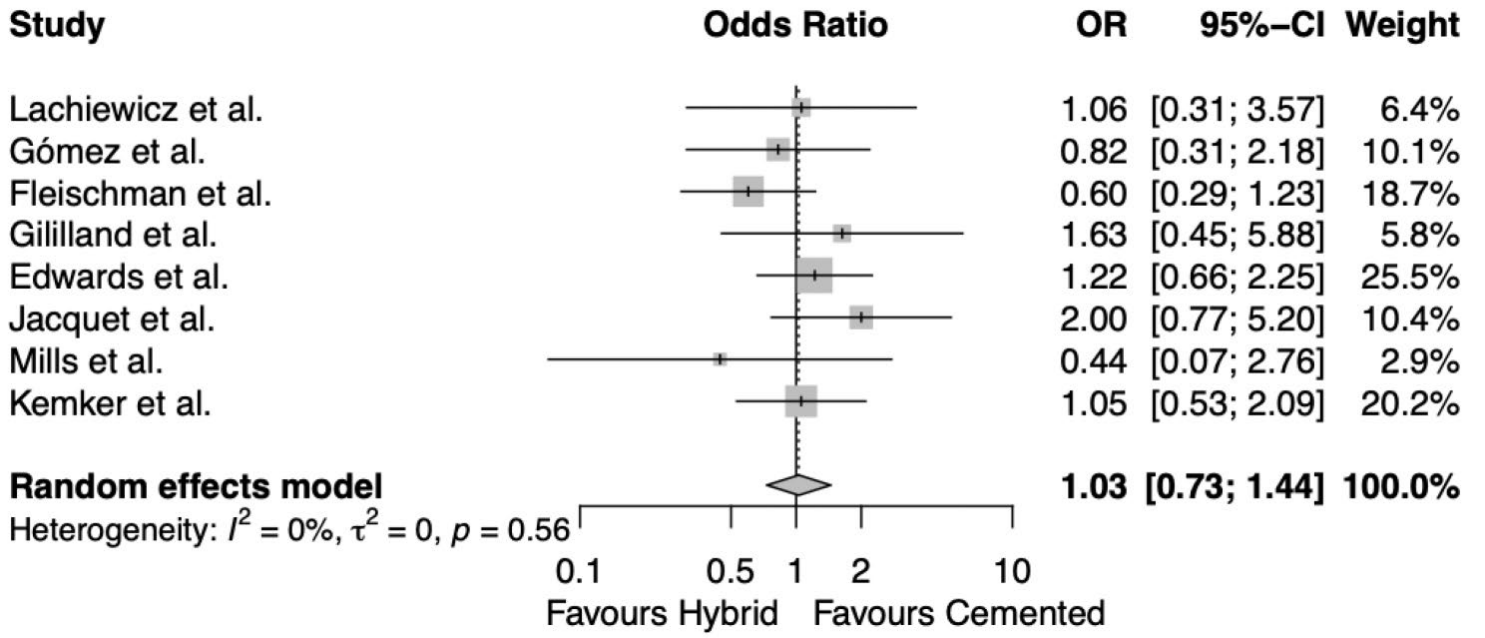
Random effects meta-analysis for total re-revision

In terms of total re-revisions across all the studies that were included, all studies reported cases of re-revisions and indications for it as well (Fig. 4). These include infection, periprosthetic fractures, implant breakage or loosening, instability, loosening, insert exchange, secondary placement of patella prosthesis and medial patellofemoral ligament reconstruction. These indications are summarized in Table 2. The random effects meta-analysis revealed that there was no difference between cemented and hybrid fixation methods in subsequent re-revision (OR 1.03, CI 0.73–1.44). There was also no significant heterogeneity (I^2^ = 0%, *p* = 0.56).

**Table 2:** Indications of re-revision

Study	Indications of Re-Revision Surgery
Lachiewicz PF et al.	- Re-operation for any reason including infection - Periprosthetic fracture - Tibial component loosening
Gómez-Vallejo J et al.	- Late onset infection - Femoral stem breakage caused by fatigue - Instability
Andrew N Fleischman et al.	- Loosening - Infection
Kosse NM et al.	- Insert exchange - Secondary placement of patella prosthesis
Heesterbeek PJ et al.	- Insert exchange - Secondary patellar resurfacing - Arthrodesis - MPFL recon
Jeremy M Gililland et al.	- Infection - Instability - Loosening - Malrotation - Peri-prosthetic fracture
Edwards PK et al.	- Recurrent infection - Aseptic loosening
Jacquet C et al.	- Infection - Dislocation - Postoperative peri-prosthetic fracture
Mills K et al.	- Neuroma - Persistent pain
Kemker BP et al.	- Any surgical re-operation e.g. explant, amputation, polyethylene exchange, lysis of adhesions, fracture fixation, extensor mechanism reconstruction - repeat revision TKA

### Aseptic re-revision

**Fig. 5:**
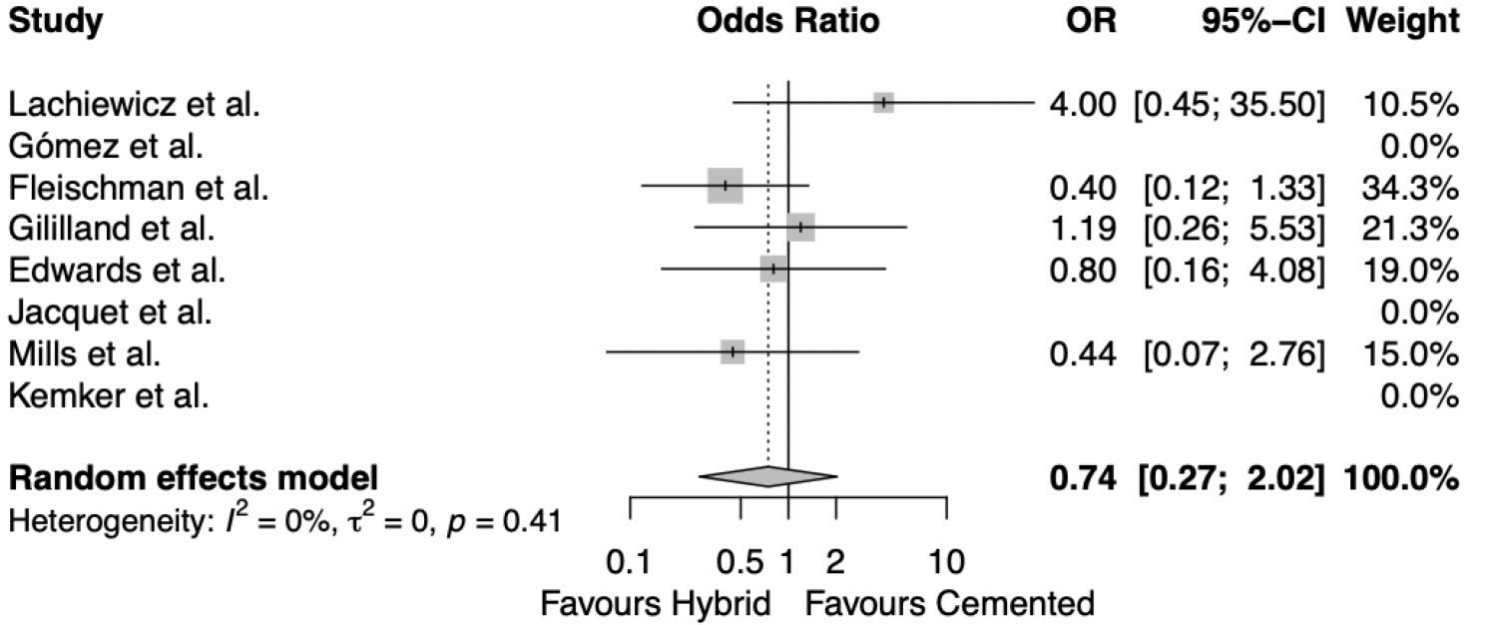
Random effects meta-analysis for aseptic re-revision

Looking at aseptic re-revision between cemented and hybrid fixation of diaphyseal stems in revision TKAs, five out of the eight papers reported cases of aseptic re-revision [16, 20, 22–24]. The random effects meta-analysis (Fig. 5) once again revealed that there was largely no difference between cemented and hybrid fixation, with results very slightly favouring hybrid fixation (OR 0.74, CI 0.27–2.02, I^2^ = 0%, *p* = 0.41).

### Other outcome measures

**Fig. 6:**
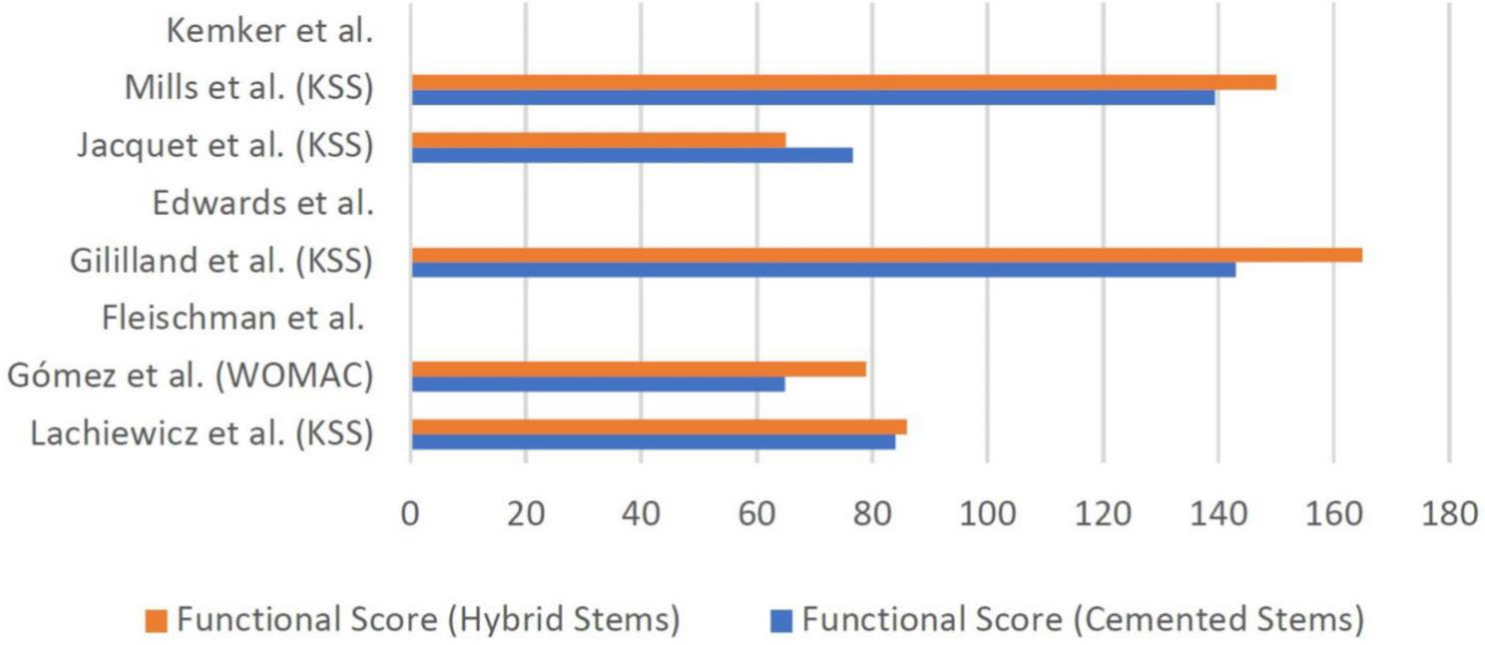
Comparison of post-operative functional scores

**Fig. 7:**
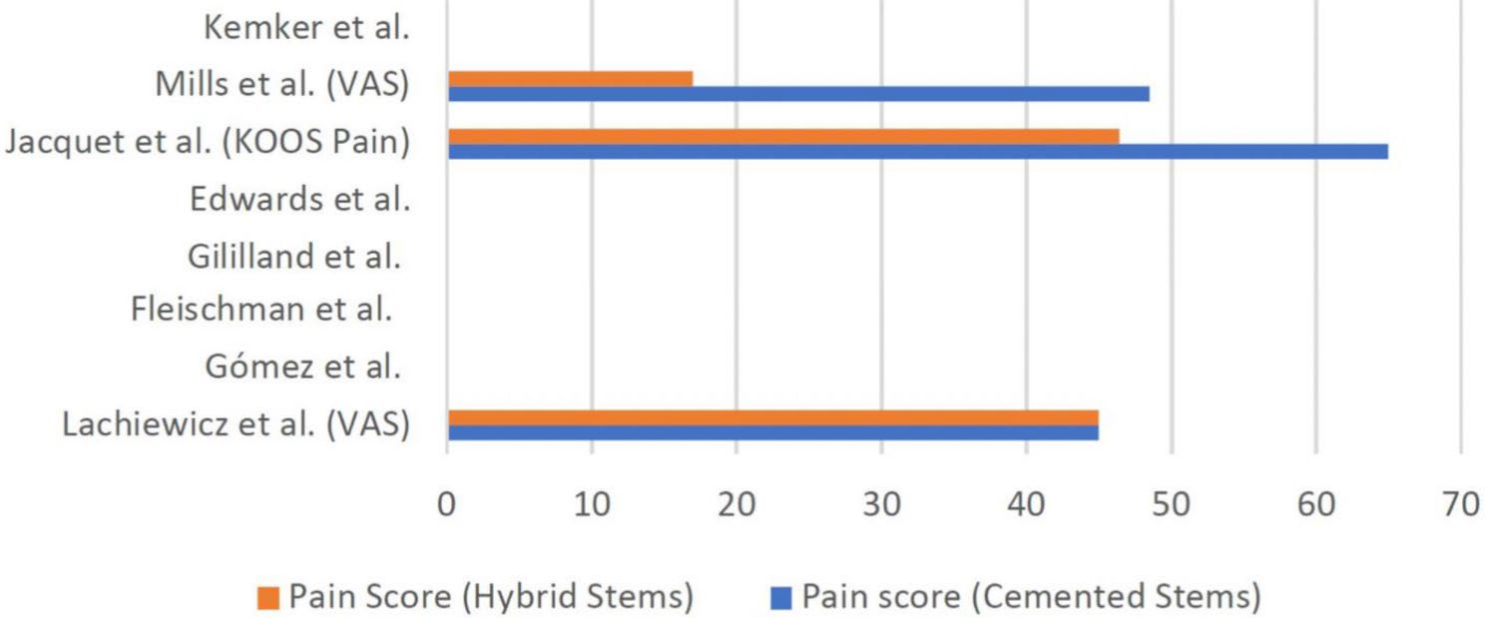
Comparison of post-operative pain scores

The authors were keen to explore how the type of stem fixation affected radiological outcomes and the incidence of re-revision. However, there were a few studies that went beyond just looking at these outcomes and looked at functional outcomes post operatively. The Western Ontario and McMaster Universities Osteoarthritis Index (WOMAC) score was used by Gomez et al., which revealed that cemented stems had a poorer score compared to hybrid stems (64.9 ± 16.8 vs. 78.9 ± 9, *p* = 0.001) [19]. The Knee Society Score (KSS) was used in all studies except for the study carried out by Fleischman et al. and Edwards et al. which did not assess functional scores, as well as Kemker et al. which also did not look into functional scores but looked more into comparing survivorship and predictors [19–22, 24]. Amongst these seven studies, the KSS was found to be similar between the two types of revision TKA techniques except for two studies. Gililland et al. found that the overall KSS was similar between the two groups, however looking at specific KSS components, the patients who received cemented revision TKAs had more improvement in clinical scores (46 ± 26 vs. 23 ± 19, *p* = 0.02) but less improvements in functional scores (10 ± 15, 22 ± 18, *p* = 0.04) compared to those who received hybrid fixation [20]. Jacquet et al. also found that, cemented stems had a more significant change in KSS scores compared to cementless stems. In terms of measuring pain, three of the eight studies investigated and reported no difference between the two groups [21, 22, 24]. Figures 6 and 7 illustrates the bar graph comparing post-operative functional and pain outcomes respectively between the two groups, across the different studies.

## Discussion

In this systematic review and meta-analysis, the authors have pooled together existing literature making a direct comparison between different stem fixation methods for revision TKAs. In the revision setting with compromised periarticular bone, it is important to achieve stable implant fixation which contribute to the durability of revised components. To enhance stability, implants with extended stems have been used to transfer stress from the deficient plateau to the shaft [26–28]. Despite the success of using intramedullary stems for revision TKAs being proven in other studies [8, 9, 29], there is paucity in the literature and controversy in the type of fixation of these stems. The most important finding of the present study that the authors aimed to investigate, is that the type of stem fixation used did not affect final clinical and radiological outcome. Both the cementless and cemented intramedullary stems fixation achieved similar stability and durability.

Diaphyseal engaging cementless stems have proven to have better alignment within the intramedullary axis and better chances of bone preservation [20]. However, studies have also shown that using hybrid stem fixation may potentially cause end of stem pain [30], although the authors recognize that other studies have shown that it can be reduced with slotted stems [31]. On the other hand, cemented stems may be easier to implant, achieve excellent initial stability, less micromotion and have additional benefits of mixing antibiotics for prolonged release. However, during subsequent re-revisions, it may potentially be more difficult to remove cemented stem without causing additional bone loss [20, 32]. Comparing between the two methods, the current literature has yet to come to a consensus as to which method is more ideal, as both methods have been proven to be effective [17, 31]. As most studies have reported and described outcomes from each individual method, there is a lack of studies directly comparing between the two.

A recent systematic review and meta-analysis carried out by Sheridan et al. also explored this controversial topic by carrying out a random-effects meta-analysis on comparative studies on outcomes between hybrid and cemented stem fixation [18]. In that study, the primary aim was to look at a combined outcome measure of “all cause failure”, defined as the summation of incidence of re-revision as well as radiological loosening. The team postulated that by doing that, there could have been a more sensitive and accurate representation of outcomes between the fixation techniques. Even though the authors recognize that that definition may encompass all factors affecting outcomes from the respective techniques, in this study, the authors have opted to use a different approach as it is unclear whether there may be potential confounders or cases being counted twice where radiologically loose cases eventually required re-revision, and how these cases may be accounted for. In that previous study, there was a statistically significant reflection that hybrid stems have lower all-cause failure than cement fixation in that regard. That was not found in this study, where the respective random effects meta-analysis for both outcome measures (radiological loosening and total re-revisions) were not significant, although there was a shift favoring hybrid stems. The focus of this study was to look at issues caused by each fixation technique, with the end point of either being deemed radiologically loose or requiring total removal of implants or any form of surgery. The authors recognize that the difference in the definitions of the outcomes may contribute to the differing results as well. In addition, the study carried out by Mills et al. which was included in this study also served as a follow on study on outcomes after ten years follow up from prior studies [33, 34]. In the study carried out by Sheridan et al., both prior studies carried out by Heesterbeek et al. and Kosse et al. was included in the analysis [18]. However, for this study, the authors intentionally omitted results carried out by the prior two studies as repeat analysis from the same population will be done, in hopes of producing more accurate results.

In this study, the authors found that there is no significant difference in radiological loosening between hybrid and cemented stems, despite results trending in favour of hybrid stems. These were results echoed in a recent meta-analysis study carried out by Wang et al. in 2015 too. In that study, Wang and his team combined studies that reported outcomes from individual fixation techniques respectively [17]. Despite not being a direct comparison between the two techniques, hybrid stems were also shown to have similar rates of failure and radiological loosening compared to cemented stems. In this study however, the authors have chosen to adopt a different methodology and chose to include only papers showing a direct comparison between cemented and hybrid stems. Despite a difference in methodology, the outcomes from both studies were largely similar. The difference in methodology could likely be one of the many factors resulting in a higher heterogeneity in the meta-analysis carried out by Wang et al. as well, especially by combining multiple studies and comparing them between the two groups.

This study has also found that rates of total re-revisions and aseptic re-revision between cemented and hybrid stems were similar between the two groups. This was a finding that was similar to studies done by both Sheridan et al. and Wang et al. [17, 18]. Despite including more papers to pool a larger sample size for comparison, and modifying the methodology, this was still a persistent finding across these three different studies. Interestingly, apart from looking at radiological and re-revision as an end-point between the two groups, most studies that explored functional and patient reported outcome measures also mostly reported that there was no difference between them. With many studies reporting hybrid stems having issues such as end of stem pain, all of the studies that compared outcomes of pain between hybrid and cemented stems reported that there was no difference between the two groups too [21, 22, 24].

Of note, the authors in this study has compared only cemented and hybrid stem fixation techniques for revision TKA. However, in the fixation of revision TKA, it is important to consider the anatomical zones as previously described by Jones et al. which consist of three main zones – namely the epiphysis, metaphysis and diaphysis, where at least two out of the three zones require good fixation [35]. In the existing literature, there is a trend moving towards the use of metaphyseal cone fixation as well due to the benefits it provides from rigid fixation of bone ingrowth after bone loss in the diaphyseal region [36–38]. In this meta-analysis, the study carried out by Jacquet et al. explored different stem fixation techniques including the use of metaphyseal cones which was not included in the pooled analyses, but the use of it with a short cemented tibial stem showed good five year follow up results compared to one combined with a long diaphyseal tibial stem or metallic augments [21]. Similar results showing the successes of metaphyseal cones were echoed in a study carried out by Bedard et al. which showed that diaphyseal impaction grafting with the use of a metaphyseal cone provided excellent radiological outcome and survivorship [39]. Future studies could investigate the outcomes comparing the use of various other fixation techniques apart from just cemented and hybrid diaphyseal stems.

## Limitations

There are several limitations in this systematic review and meta-analysis. Firstly, despite including as many recent studies as possible in the overall analysis, there were very few studies with high levels of evidence apart from one randomized controlled trials of long term follow up which stemmed from the same pool of patients extended across different follow up periods [24]. However, the authors recognize that it is difficult to ethically justify carrying out randomized controlled trials surrounding this topic especially with the controversy in outcomes in the literature between the two groups, which this study aims to address. Secondly, there was an issue with missing data in several studies, as well as heterogeneity when comparing studies together, which was an inevitable problem and was difficult to reconcile when carrying out the analysis. The authors have attempted to address this by assessing the I^2^ in every forest plot to ensure minimal heterogeneity affecting the study, which was found to not be statistically significant. In a similar vein, other missing information such as the type of polyethylene insert used, implant designs adopted (including the use of metaphyseal fixation devices or length of diaphyseal stems), intra-operative assessment of bone loss and use of augmentation devices were not reported in all studies which can also potentially alter the results of this study and contribute to heterogeneity.

## Conclusion

In conclusion, this study has found that there is no one technique that is superior to the other despite hybrid stems seemingly showing a slight edge over cemented stems in terms of radiological failure and loosening. The authors would nonetheless recommend considering other factors such as surgeon and patient comfort level in carrying out these techniques as well as the potential chance for re-revision again especially in cases such as prosthetic joint infections. Future studies should also assess functional, and patient reported outcome measures levels further to give us a better understanding on patient experience post-operatively.

## Data Availability

We propose a systematic review and meta-analysis. All articles where data were extracted from are listed in the summary tables included in the manuscript.
